# Decreased Variability of the 6-Minute Walk Test by Heart Rate Correction in Patients with Neuromuscular Disease

**DOI:** 10.1371/journal.pone.0114273

**Published:** 2014-12-05

**Authors:** Kira P. Prahm, Nanna Witting, John Vissing

**Affiliations:** Neuromuscular Research Unit, Department of Neurology, Rigshospitalet, University of Copenhagen, Denmark; Charité Universitätsmedizin Berlin, NeuroCure Clinical Research Center, Germany

## Abstract

**Objective:**

The 6-minute walk test is widely used to assess functional status in neurological disorders. However, the test is subject to great inter-test variability due to fluctuating motivation, fatigue and learning effects. We investigated whether inter-test variability of the 6MWT can be reduced by heart rate correction.

**Methods:**

Sixteen patients with neuromuscular diseases, including Facioscapulohumeral muscular dystrophy, Limb-girdle muscular dystrophy, Charcot-Marie-Tooths, Dystrophia Myotonica and Congenital Myopathy and 12 healthy subjects were studied. Patients were excluded if they had cardiac arrhythmias, if they received drug treatment for hypertension or any other medical conditions that could interfere with the interpretation of the heart rate and walking capability. All completed three 6-minute walk tests on three different test-days. Heart rate was measured continuously.

**Results:**

Successive standard 6-minute walk tests showed considerable learning effects between Tests 1 and 2 (4.9%; *P* = 0.026), and Tests 2 and 3 (4.5%; *P* = 0.020) in patients. The same was seen in controls between Tests 1 and 2 (8.1%; *P* = 0.039)). Heart rate correction abolished this learning effect.

**Conclusion:**

A modified 6-minute walk test, by correcting walking distance with average heart rate during walking, decreases the variability among repeated 6-minute walk tests, and should be considered as an alternative outcome measure to the standard 6-minute walk test in future clinical follow-up and treatment trials.

## Introduction

The 6-minute walk test (6MWT) is a submaximal exercise test, which despite pitfalls, is widely used to assess treatment efficacy or disease progression in neurological diseases, because walking capability is a central function of life and reflects an important functional capacity of daily living [1], [2]. However, the test is subject to a high inter-test variability, related to fluctuating motivation, fatigue, and learning effects [3]–[8]. There is great need for outcome measures in clinical trials, which are sensitive enough to detect even small changes of function, and as such, the 6MWT is rather insensitive in its present form.

Cardiac output (CO) is known to be linearly correlated to work load, irrespective of fatigue and motivation, and since heart rate (HR) is a function of CO (CO  =  stroke volume x HR) [9], [10], we hypothesized that a correction of the 6MWT distance by average HR can smooth out day-to-day variability of the test due to motivation or fatigue on the day of testing, or due to a learning effect. Moreover, reliability and feasibility of the 6MWT, for adult patients, has only been validated in one other study of neuromuscular disorders (NMD) [2]. We, therefore, wanted to test if the learning effect after repeated 6MWTs is present in a broad group of patients with NMD, if their walking distance correlates with HR, and if HR correction of the 6MWT can reduce inter-test variability.

## Methods

### Subjects

The study included 16 patients, recruited from our neuromuscular clinic, and 12 healthy subjects. All diagnoses in patients were verified by genetic testing. Some had preferential proximal and other distal lower limb weakness (Table 1). Inclusion criteria were; ability to walk 60 meters, and left ventricular ejection fraction above 40%. Exclusion criteria were arrhythmias, drug treatment that could affect blood pressure and HR, and other medical conditions that could interfere with the interpretation of the HR and walking capability. The reason for including a control group was to identify, if a learning effect for the 6MWT, which has formerly been described in several studies of healthy subjects, was similarly present in both controls and patients with neuromuscular disorders. Furthermore, we wanted to investigate if a correlation between walked distances and mean HR existed. And if it was present in both patients and controls, we wanted to test whether HR correction was feasible in both patients and healthy controls. Oral and written informed consent was obtained from all subjects, and the study was approved by the Regional Committee on Health Research Ethics in Denmark.

**Table 1:** Gene mutations and muscle strength in 16 patients with neuromuscular disorders.

			MRC	MRC	MRC
Patient	Disease	Age	Hip (F/E)	Knee (F/E)	Ankle (F/E)
1	LGMD2I	60	R(1/4); L (1/4)	R(4/4); L(4/4)	R(5/5); L(5/5)
2	LGMD2I	52	R(2/4); L (2/4)	R(2/4); L (2/4)	R(5/5); L(5/5)
3	LGMD2I	20	R(5/5); L(5/5)	R(5/5); L(5/5)	R(5/5); L(5/5)
4	LGMD2A	63	R(4/4+); L(4/4+)	R(4/5); L(4/5)	R(5/5); L(5/5)
5	DM 2	64	R(5/5); L(5/5)	R(5/5); L(5/5)	R(4/4+); L(4/4+)
6	DM2	39	R(5/5); L(5/5)	R(5/5); L(5/5)	R(5/5); L(5/5)
7	CMT1A	63	R(5/5); L(5/5)	R(5/5); L(5/5)	R(4-/5); L(4-/5)
8	CMT1A	47	R(5/5); L(5/5)	R(5/5); L(5/5)	R(4/5); L(4/5)
9	CMT1A	52	R(5/5); L(5/5)	R(5/5); L(5/5)	R(4-/5); L(4-/5)
10	CM	21	R(4/4); L(4/4)	R(4/4); L(4/4)	R(4/4); L(4/4)
11	CM	54	R(4-/4-); L(4-/4-)	R(4-/4-); L(4-/4-)	R(1/2); L(1/2)
12	FSH	60	R(5/5); L(5/5)	R(5/5); L(5/5)	R(5/5); L(5/5)
13	FSH	31	R(5/5); L(5/5)	R(5/5); L(5/5)	R(5/5); L(5/5)
14	FSH	50	R(5/5); L(5/5)	R(5/5); L(5/5)	R(4/5); L(4/5)
15	FSH	36	R(5/5); L(5/5)	R(5/5); L(5/5)	R(4/5); L(4/4)
16	FSH	46	R(5/5); L(4/5)	R(5/5); L(5/5)	R(4/5); L(4+/5)

Abbreviations: LMGD  =  Limb-girdle muscular dystrophy, DM  =  Myotonic dystrophy, CMT  =  Charcot-Marie-Tooth disease, CM  =  Congenital myopathy, FSH  =  Facioscapulohumeral muscular dystrophy, MRC  =  Medical Research Council Scale, (F/E)  =  Flexion/Extension, R =  right, L =  left.

### Procedures

Each subject completed three 6MWTs, on three different test-days, one week apart. On each test-day the test subjects performed one 6MWT according to the American Thoracic Society guidelines (ATS) (Table 2) [1]. Subjects were asked to avoid heavy meals 2 h before testing, and caffeine, alcohol, and hard physical exercise within the last 24 h. Before each test, subjects were told to walk as far as possible during the 6 minutes, not to speak, that rests were allowed, but that they should resume walking as soon as they were able. During the 6MWT, patients were given feedback at the end of every minute with encouragement such as “you are doing well”. HR was measured continuously every second during walking, by a pulse-watch (Polar, Heart rate monitor, Finland) (File S1, File S2), and the mean of measured heart rate during each walking period was used for analysis. For consistency, the same investigator conducted all tests.

**Table 2:** Walked distance during each test for patients and controls.

	Test 1	Test 2	Test 3		
Patient				Mean	SD
1	420	419	424	421	2.6
2	189	186.2	199	191.4	6.7
3	735	754	780	756.4	22.4
4	315	323	318	318.7	4
5	515	549	557	540.3	22.3
6	343	356	368	355.7	12.5
7	498	614	720	610.7	111
8	484	515	553	517.3	34.6
9	434	448	468	450	17.1
10	678	654	680	670.7	14.5
11	531	559	567	552.3	18.9
12	611	571	659	613.7	44.1
13	646	743	795	728	75.6
14	535	572	577	561.3	22.9
15	538	561	527	542	17.3
16	305	334	329	322.7	15.5
**Min**	189	186.2	199		
**Max**	735.3	754	795		
**Mean**	486.1	509.9	532.6		
**SD**	147.4	156.2	173.0		
**Control**	**Test1**	**Test 2**	**Test 3**		
1	679	772	751.5	734.2	48.9
2	749	893.2	839	827.1	72.8
3	962	953	900	938.3	33.5
4	641	850	935	808.7	151.3
5	695	725	774	731.3	39.9
6	735	664	670	689.7	39.4
7	705	705	655	688.3	28.9
8	675	705	740	706.7	32.5
9	645	674	733	684	44.8
10	788	836	839	821	28.6
11	660	658	667	734.2	4.7
12	750	965	973	896	126.5
**Min**	641	658	655		
**Max**	962	965	973		
**Mean**	723,7	783,4	789,7		
**SD**	87,9	112,3	107,3		

### Statistics

Results are expressed as means ± standard deviations (SD), or average change in percentage and range. Demographic data between patients and controls were compared by an unpaired two-sample student's t-test. A statistical significance of differences between mean 6MWT distances was assessed by a one-way ANOVA of repeated measures with a Greenhouse-Geisser correction. Pairwise comparisons of the specific differences in the 6MWT distances were subsequently assed by the Bonferroni post hoc test. Pearson's Correlation was used to investigate whether there was an association between walked distance and HR. Statistical significance was defined by *P* value ≤0.05.

All statistical analyses were performed using IBM SPSS statistical software version 20.0, and Microsoft Excel version 14.4.3, 2011.

## Results

### Characteristics of the study population

Except for age, demographic data were comparable between patient and healthy subjects (Table 3). Muscle affection was primarily proximal in LGMD patients, distal in patients with CMT1A and FSHD, and diffuse in patients with congenital myopathy and myotonic dystrophy type 2 (Table 1).

**Table 3:** Characteristics of patients and controls.

	Patients		Controls	
	n = 16		n = 12	
	Mean (SD)	Min-Max	Mean (SD)	Min-Max
Sex (M/F)	(7/9)		(6/6)	
Age (years)	47.4 (14.4)*	20–64	34.8 (14.5)*	20–67
Height (m)	173.3 (9.6)	158–187	175 (6.8)	166–189
Weight (kg)	75.2 (11.8)	57–100	73.8 (23.2)	53–235
BMI (kg/m2)	25.1 (3.5)	20.2–30.5	23.7 (5.5)	19.2–37.8

M =  male, F =  female, BMI  =  body mass index. *P<0.05.

### 6MWT: Inter-test variation and effects of correcting with heart rate (Figure 1)

A repeated measures ANOVA with a Greenhouse-Geisser correction determined that mean walking distance differed significantly between time points for both patients (*F*(1.335, 20.026)  = 8.262, *P* = 0.006) and controls (*F*(1.248, 13.727)  = 4.417, *P* = 0.048). Post hoc tests using Bonferroni correction for sequential 6MWTs showed a considerable learning effect in patients, with 24 m (4.9%; *P* = 0.026) between Tests 1 and 2, and 23 m (4.5%; *P* = 0.020) between Tests 2 and 3. The same was seen in controls (59 m between Tests 1 and 2 (8.1%; *P* = 0.039)). No significant difference was observed between Test 2 and Test 3 for controls.

**Figure 1:**
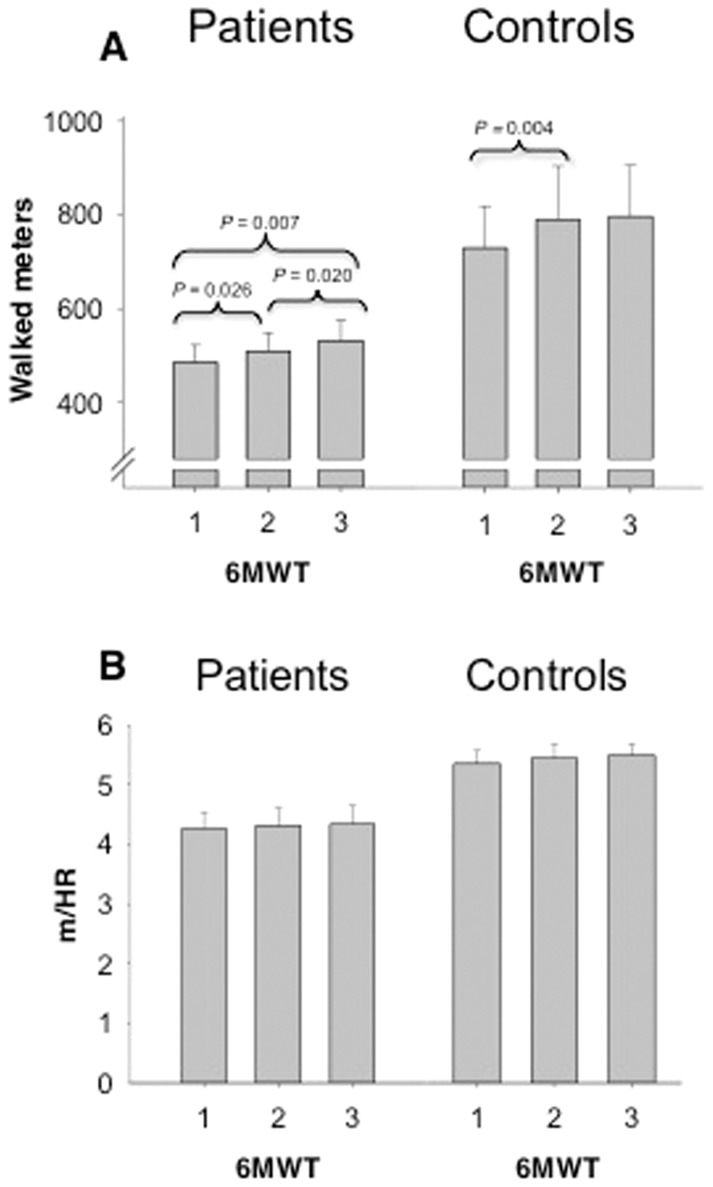
Distance walked with, and without heart rate correction. A. Mean distance walked in all 6-minute walk tests, for both patients and controls. The graph shows the significant increases, marked by the *p*-value, in mean walked distance among tests. B. Heart rate corrected 6-minute walk distance in all 6-minute walk tests, for both patients and controls. No significant difference between walked distances was observed.

**Figure 2:**
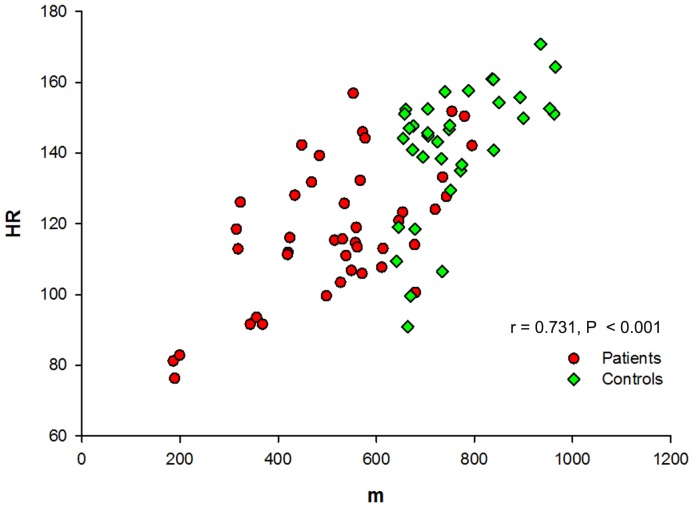
Correlation between heart rate and 6-minute walk distance. The graph shows the Pearson's correlation between heart rate and walked distance for both patients and controls.

Pearson's Correlation showed that walking distance and HR correlated well (*r*  = 0.731; 95% confidence interval: 0.573–0.886, *P*≤0.001) in the 28 subjects (Figure 2). Thus, when correcting distance in 6MWT by HR, all day-to-day variations were abolished (Figure 1B).

## Discussion

This study shows that a 6MWT corrected for HR has much less inter-test variability, and is therefore a more consistent outcome measure to assess walking ability in NMDs and healthy subjects than a standard 6MWT. Thus, HR-adjusted walking distance corrects day-to-day variation in the 6MWT, due to learning effects, motivation, and fatigue. Such pitfalls are otherwise well acknowledged limitations of the test in a number of diseases [10]. Our study also shows that a learning effect is present in a wide range of different NMDs, similar to the learning effect shown in earlier studies of other diseases [3]–[8], which cautions the use of the standard 6MWT in uncontrolled trials of NMDs.

The reliability of the 6MWT in adult patients with NMD has only been validated in patients with myotonic dystrophy type 1, in whom an increase in walking distance was observed with repeated 6MWTs [2]. Practice tests were therefore recommended.

The overall close correlation between HR and distance walked in the standard 6MWT was also found for repeated tests in individual subjects with the exception of three patients and two controls. There was no obvious explanation for the absence of correlation in these subjects, but HR responses may have been influenced by use of tobacco, alcohol or caffeine before experiments, although subjects were told to refrain from such use. However, our results demonstrate that in larger studies, a few patients in whom a coupling between HR and walking distance is disturbed, will not distract from the main conclusion, that HR-adjusted walking distance is a stronger outcome measure than a “raw” walking distance. Nevertheless the HR-adjusted 6MWT should only be used in patients without cardiac affection. Our findings can likely be extended to other diseases in which the 6MWT is commonly used, as the source of day-to-day variation of the test (learning effects, motivation, fatigue) is very similar across diseases. However, studies are necessary to corroborate this in individual diseases. Heart rate is an easily measured clinical parameter, and analysis of the linear correlation between walked meters and mean measured HR can easily be obtained for follow-up assessments and as endpoint in clinical trials, in which 6MWT is used as efficacy parameter. 6MWT corrected by mean HR during the test (walked meters/mean HR), likely can detect clinically meaningful changes in a trial, as indicated by a longer distance walked at a similar HR on treatment or a similar distance walked at a lower HR. However, this relationship should be confirmed in future treatment studies, to verify that the HR-corrected 6MWT, which abolishes the confounding factors from a learning effect, can also detect treatment effects.

## Conclusion

Our findings suggest that a heart rate-corrected 6MWT is a more accurate outcome measure than the standard 6MWT, and should be considered as a future outcome measure of walking capability.

## Supporting Information

File S1 **Continued measured heart rate data for controls.**
(XLSX)

File S2 **Continued measured heart rate data for patients.**
(XLSX)
